# Attempted Suicide by Massive Warfarin Ingestion Conservatively Managed Using Phytonadione

**DOI:** 10.1155/2016/7095251

**Published:** 2016-12-04

**Authors:** Katherine L. March, Kruti S. Patel, Jennifer D. Twilla

**Affiliations:** ^1^Methodist University Hospital, 1265 Union Ave., Memphis, TN 38104, USA; ^2^Department of Clinical Pharmacy, University of Tennessee Health Science Center, 881 Madison Ave., Memphis, TN 38163, USA; ^3^Department of Medicine, University of Tennessee Health Science Center, 910 Madison, Suite 1002, Memphis, TN 38163, USA

## Abstract

Treatment strategies for acute toxicity following massive ingestion of warfarin are not well described in the literature. Warfarin is the primary oral anticoagulation agent used in the treatment of thromboembolic disease, and patients with acute toxicity are at risk for life-threatening hemorrhages. Treatment options include phytonadione (vitamin K_1_), fresh frozen plasma (FFP), and prothrombin complex concentrates (PCCs) used alone or in combination. FFP and PCC can be associated with volume complications, undesirable thromboembolic events, and increased costs. We describe the case of a 63-year-old female with acute warfarin toxicity following a massive ingestion of warfarin (420 mg–450 mg) in an attempt to commit suicide. Upon arrival to the emergency department, serial INR checks were initiated to help guide dosing strategy and later adjusted based on INR response to treatment using only phytonadione.

## 1. Introduction

While the use of new oral anticoagulation agents is on the rise, the vitamin K antagonist (VKA) warfarin remains a primary agent for oral anticoagulation in the treatment of thromboembolic disease [1]. Due to its extensive interpatient variability and narrow therapeutic range, warfarin requires frequent laboratory monitoring with International Normalized Ratio (INR) testing and close patient follow-up [2]. Without these efforts, patients can experience warfarin toxicity due to a multitude of reasons including dose changes, drug-drug interactions, and dietary changes [2]. Additionally, although uncommon, intentional ingestion of large doses of warfarin can lead to life-threatening acute toxicity scenarios.

Acute warfarin toxicity management is complicated by its well described pharmacokinetic profile including rapid and complete absorption, delayed anticoagulation effects, and having a 29- to 45-hour half-life [3, 4]. Various guidelines are currently available to aid in supratherapeutic INR management but do not specifically address acute overdose management [5, 6]. Phytonadione (vitamin K) remains the most commonly used first-line reversal agent for VKA therapy in patients presenting with a supratherapeutic INR with or without significant bleeding [7]. Additionally, fresh frozen plasma (FFP) and prothrombin complex concentrates (PCCs) have also been proven effective in the reversal of warfarin [8, 9]. Although they are effective at warfarin reversal, each can be associated with undesirable complications. When compared to PCC, the administration of plasma has been associated with a greater risk of volume overload compared to PCC products [10]. In contrast, while PCC is not associated with volume overload complications it does pose a risk of creating unwanted thromboembolic events [11].

To date, there are only a limited number of cases reports available describing massive warfarin ingestion (>300 mg) [12–15]. Here we describe a case of acute warfarin toxicity following a massive ingestion of warfarin (420 mg–450 mg) in an attempt to commit suicide that was conservatively managed using only phytonadione.

## 2. Case Presentation

A 63-year-old Caucasian female was brought to our emergency department (ED) after ingesting approximately 84 to 90 tablets of warfarin 5 mg (420 mg–450 mg), 6 tablets of mycophenolate mofetil 500 mg, an estimated 62 tablets of gabapentin 300 mg, and an unknown amount of over-the-counter sleeping pills. Pill counts were reported by the patient or estimated based upon the pharmacy fill date on the patient's prescription bottles and the day of intentional ingestion. All three pill bottles were found empty next to the patient after the self-reported ingestion in an attempt to commit suicide. The patient had a past medical history significant for hypertension, large vessel cerebral vascular disease, stroke, neurovasculitis, pulmonary embolism (PE), deep vein thrombosis (DVT), inferior vena cava filter placement, and depression. Her home medication regimen included lisinopril 10 mg, pantoprazole 40 mg, topiramate 25 mg, and venlafaxine 75 mg, all taken once daily, and gabapentin 600 mg, mycophenolate mofetil 500 mg, and ranitidine 150 mg, all taken twice daily, as well as clonazepam 1 mg and pramipexole 0.125 mg taken daily at bedtime. Her home warfarin regimen was 5 mg on Sunday, Tuesday, Thursday, and Saturday and 7.5 mg on Monday, Wednesday, and Friday.

Upon her initial presentation to the ED, the patient was extremely drowsy but responded to tactile and painful stimuli. Vital signs at time of admission were as follows: blood pressure (BP) 176/120, heart rate (HR) 86 beats per minute (bpm), respiratory rate (RR) 18 breaths per minute, 100% oxygen saturation, and a temperature of 36.3°C. Other pertinent lab tests at admission included a hemoglobin of 13.8 g/dL, hematocrit of 42.5%, an INR of 2.8, and a prothrombin time (PT) of 29.1 seconds. An arterial blood gas (ABG) test performed in the ED showed the patient was acidotic with a blood pH of 7.3, bicarbonate level of 22, and a base excess of negative 5. Her partial carbon dioxide (pCO_2_) was within normal limits (WNL). Electrolytes were WNL with the exception of mild hypokalemia (3.4 mEq/L) and mild hyperchloremia (108 mmol/L). An initial computed tomography (CT) scan of the brain was performed to look for any intracranial hemorrhaging which showed no acute findings. The patient was subsequently transferred to the ICU, and, at the recommendation of the Poison Control Center, serial INR checks were initiated at 12-hour intervals.

**Figure 1:**
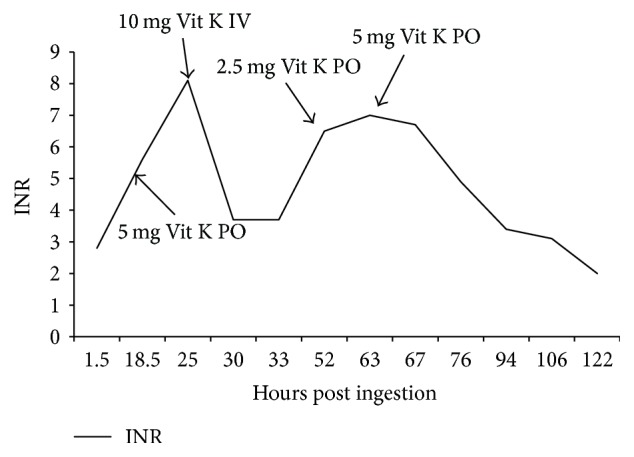
Patient's INR trend during course of hospitalization.

On day 1 of hospitalization the patient's mentation began to improve and she reported no thoughts of suicidal ideation. As anticipated, the INR began to trend upward first to 5.6 before peaking at 8.1 on day 1.  Figure 1 depicts all INR values and times drawn as well as phytonadione doses and times administered during the course of hospitalization. After the first INR of 5.6 the patient received a one-time 5 mg oral dose of phytonadione. When the INR of 8.1 was reported she was then ordered to receive another one-time 10 mg dose of phytonadione via an IV piggyback. Mycophenolic acid and mycophenolic acid glucuronide levels were also reported on day 1 of hospitalization at 4.6 mcg/mL and 150 mcg/mL, respectively. Other lab tests on day 1 were WNL except for a potassium level of 6.1 mEq/L.

Her INR remained stable at 3.7 until late on day 2 at which time it rose to 6.5. A one-time 2.5 mg oral dose of phytonadione was administered following this increase. The INR peaked at 7 before trending down to 6.7 and 4.9 on day 3. An additional single 5 mg oral dose of phytonadione was given following the reported INR of 7. Following this dose of phytonadione, the INR continued to trend downward until day 6 when the patient was discharged with an INR of 2.

Throughout the course of hospitalization, the patient's lab tests remained WNL except where otherwise noted and she did not exhibit any signs or symptoms of bleeding. On day 3 of hospitalization the patient complained of headaches in the frontal region for which a CT scan of the brain was performed which ruled out acute bleeding. The patient was examined by behavioral health services and was deemed mentally competent to return home prior to discharge. She was discharged home on her previous medication regimen except that she was instructed to hold her warfarin therapy. A follow-up appointment was arranged with her primary care physician prior to discharge.

## 3. Discussion

Vitamin K antagonists have been the drug of choice for the treatment of thromboembolic disease since their discovery in the mid-1900s. Intentional consumption of massive doses of warfarin is a rare clinical scenario. When healthcare professionals are presented with such a case, it can be perplexing and complicated to manage. A retrospective chart review conducted by Lousberg et al. demonstrated that less conservative management strategies with phytonadione, FFP, and PCC can lead to episodes of major bleeding; however, the authors also showed overly aggressive treatment of anticoagulation reversal with the aforementioned agents can lead to iatrogenic thromboembolic complications [16]. For chronic warfarin users, a fine balance exists between preventing major bleeding from excessive anticoagulation and complete coagulation reversal.

Warfarin is a racemic mixture of R and S enantiomers which are rapidly absorbed from the GI tract and have high bioavailability and half-lives ranging from 29 to 45 hours [3, 4]. Maximal serum concentrations can be seen approximately 90 minutes after ingestion [3, 4]. Warfarin exhibits its anticoagulation effects by inhibiting a vitamin K dependent carboxylation step during synthesis of clotting factors II, IV, IX, and X, as well as inactivating proteins C and S. By administering vitamin K during warfarin toxicity, these anticoagulation effects can be reversed by providing vitamin K needed to carboxylate the clotting factors. This combined with its ease of administration and low cost makes it an ideal treatment of warfarin toxicity.

In 1981, Toolis et al. described a case of massive warfarin ingestion in which a man with a prosthetic heart valve took 300 mg of warfarin in combination with alcohol. He was successfully treated over the course of six days by monitoring his prothrombin time ratio (PTR) and keeping it in a therapeutic range using repeated 300 mL infusions of FFP [12]. One adolescent male was brought to an emergency department in 2002 after intentionally ingesting 350 mg of warfarin [14]. He was treated with 10 mg of intravenous (IV) phytonadione on day one of his hospitalization but was later given two doses of FFP when his INR began to peak at 5 and 4.5 on days 3 and 4 of hospitalization, respectively. One final 10 mg dose of phytonadione was given 12 hours after the last administration of FFP at which time his INR continued to trend downward. Finally, one female presented to an emergency department in 2004 after intentional ingestion of 540 mg of warfarin [15]. At six hours after ingestion, her INR had risen to 5.1 and she was subsequently treated with 10 mg IV vitamin K, recombinant factor VIIa, and 3-factor PCC. Six hours after these interventions her INR had fallen to 0.5. She was then ordered recombinant VIIa and 3-factor concentrates as needed for an INR of greater than 5 and a scheduled regimen of vitamin K 5 mg by mouth every six hours. This was eventually decreased to 5 mg three times daily and was discontinued upon her discharge from the hospital. In all three of these cases, no bleeding or adverse events were reported.

Given the paucity of information available discussing intentional overdoses of warfarin and varying management strategies, we describe a case of acute warfarin toxicity after massive warfarin ingestion in an attempt to commit suicide. While similar to the aforementioned cases, her treatment strategy was more conservative than those previously mentioned. Massive warfarin ingestion can be an alarming scenario for healthcare professionals especially if clinical signs and symptoms of bleeding are present. While the emergence of bleeding should result in more aggressive treatment strategies with phytonadione and FFP or PCC administration, this case demonstrates if no signs or symptoms of bleeding are present, conservative management of acute warfarin toxicity is manageable using serial INRs to individualize and guide dosing strategies with oral and IV phytonadione. By utilizing this conservative management, it is possible to avoid not only unnecessary volume and thromboembolic complications that may be associated with FFP and PCC but additional costs as well.

## References

[1] Barnes G. D., Lucas E., Alexander G. C., Goldberger Z. D. (2015). National trends in ambulatory oral anticoagulant use. *The American Journal of Medicine*.

[2] Ansell J., Hirsh J., Dalen J. (2001). Managing oral anticoagulant therapy. *Chest*.

[3] Hirsh J., Dalen J. E., Anderson D. R. (2001). Oral anticoagulants: mechanism of action, clinical effectiveness, and optimal therapeutic range. *Chest*.

[4] Mephyton (Phytonadione) Vitamin K_1_, Aton Pharma, Lawrenceville, NJ, USA, 2008

[5] Holbrook A., Schulman S., Witt D. M. (2012). Evidence-based management of anticoagulation therapy: antithrombotic therapy and prevention of thrombosis, 9th ed: American College of Chest Physicians evidence-based clinical practice guidelines. *Chest*.

[6] Baker R. L., Coughlin P. B., Salem H. H., Gallus A. S., Harper P. L., Wood E. M. (2004). Warfarin reversal: consensus guidelines, on behalf of the Australasian Society of Thrombosis and Haemostasis. *Medical Journal of Australia*.

[7] Van Berkel M., Crannage A., Murphy J. (2013). Evaluation of education on the appropriate use of vitamin K in warfarin reversal in adult inpatients. *Hospital Pharmacy*.

[8] Milling T. J., Refaai M. A., Sarode R. (Apr 2016). Safety of a four-factor prothrombin complex concentrate versus plasma for vitamin K antagonist reversal: an integrated analysis of two phase iiib clinical trials. *Academic Emergency Medicine*.

[9] Milling T. J., Refaai M. A., Goldstein J. N. (2016). Thromboembolic events after vitamin K antagonist reversal with 4-factor prothrombin complex concentrate: exploratory analyses of two randomized, plasma-controlled studies. *Annals of Emergency Medicine*.

[10] Refaai M. A., Goldstein J. N., Lee M. L. (2015). Increased risk of volume overload with plasma compared with four-factor prothrombin complex concentrate for urgent Vitamin K antagonist reversal. *Transfusion*.

[11] Sin J. H., Berger K., Lesch C. A. (2016). Four-factor prothrombin complex concentrate for life-threatening bleeds or emergent surgery: a retrospective evaluation. *Journal of Critical Care*.

[12] Toolis F., Robson R. H., Critchley J. A. J. H. (1981). Warfarin poisoning in patients with prosthetic heart valves. *British Medical Journal*.

[13] Hackett L. P., Ilett K. F., Chester A. (1985). Plasma warfarin concentrations after a massive overdose. *Medical Journal of Australia*.

[14] Ramanan A. V., Gissen P., Bose-Haider B. (2002). Intentional overdose of warfarin in an adolescent: need for follow up. *Emergency Medicine Journal*.

[15] Matthews S. S., Ringeisen A. L., Wedro B. (2014). Intentional overdose of warfarin in an adult: anticoagulant reversal in the ED. *The American Journal of Emergency Medicine*.

[16] Lousberg T. R., Witt D. M., Beall D. G., Carter B. L., Malone D. C. (1998). Evaluation of excessive anticoagulation in a group model health maintenance organization. *Archives of Internal Medicine*.

